# Plasmonic Functionality of Optical Fiber Tips: Mechanisms, Fabrications, and Applications

**DOI:** 10.3390/ma16093596

**Published:** 2023-05-08

**Authors:** Bobo Du, Yunfan Xu, Lei Zhang, Yanpeng Zhang

**Affiliations:** Key Laboratory of Physical Electronics and Devices of Ministry of Education and Shaanxi Key Laboratory of Information Photonic Technique, School of Electronic Science and Engineering, Xi’an Jiaotong University, Xi’an 710049, China

**Keywords:** optical fiber, fiber tip, plasmonic functionality

## Abstract

Optical fiber tips with the flat end-facets functionalized take the special advantages of easy fabrication, compactness, and ready-integration among the community of optical fiber devices. Combined with plasmonic structures, the fiber tips draw a significant growth of interest addressing diverse functions. This review aims to present and summarize the plasmonic functionality of optical fiber tips with the current state of the art. Firstly, the mechanisms of plasmonic phenomena are introduced in order to illustrate the tip-compatible plasmonic nanostructures. Then, the strategies of plasmonic functionalities on fiber tips are analyzed and compared. Moreover, the classical applications of plasmonic fiber tips are reviewed. Finally, the challenges and prospects for future opportunities are discussed.

## 1. Introduction

Among various optical components, optical fibers possess unique properties, such as small size, mechanical robustness, flexibility, and immunity to electromagnetic interference. The tremendous success of optical fiber technology completely changes our modern society. Despite the countless applications of optical fibers around the world, the availabilities of materials (typically SiO_2_) and geometries of optical fibers obstruct further progress. The functionalization of optical fibers opens up a new pathway to extend the potential of optical fibers, launching the recent rise of “lab-on-fiber” technology [1,2]. In terms of lab-on-fiber technology, functionalized materials and components defined at the micro and nano scales are introduced to optical fiber platforms, transforming conventional optical fibers into multifunctional devices.

Basically, lab-on-fiber technology includes “lab-around-fiber”, “lab-in-fiber”, and “lab-on-tip”, where the functionalization is processed on side-walls, inner holes, and end-facets of fibers, respectively. Relatively, fiber tips are ready for integrations of functional media and plug-and-play capability due to the planar substrate and light transmission output [3,4]. However, the fiber tips suffer from short light-matter interaction distance, small sensing area, and ultra-large aspect ratio. Fortunately, the emergences of novel physical mechanisms and advanced nanofabrication techniques help address the challenges with respect to lab-on-tips.

The plasmonic phenomenon involves the strong light-matter interaction in metallic media [5,6]. The combination of plasmonics with the established fiber tips led to an exciting spectrum of possibilities, including high performances and multi-functionalities. On the other hand, the significant advances in tip-compatible nanofabrication techniques provide diverse opportunities to construct on-demand fiber tips, including plasmonic fiber tips.

In this review, we aim to provide a general overview of fiber tip devices, with a special focus on plasmonic fiber tips. Firstly, the basic physics of different plasmonic structures (planar film, isolated nanoparticle, and nano-array) are discussed in order to suggest tip-compatible plasmonic configurations. Secondly, the main fabrication techniques (self-assembly, lithography, and transfer methods) of plasmonic fiber tips are reviewed with a comparison of the merits of each technique. Furthermore, the interesting applications of plasmonic fiber tips are highlighted. Finally, challenges and prospects that lie ahead are outlined in order to motivate and direct future research efforts into plasmonic fiber tips.

## 2. Physical Mechanisms

The plasmonic phenomenon commonly refers to the optical resonances in metallic structures under external light excitations [7]. According to the excitation conditions and supporting structures, plasmonic resonances include surface plasmon resonance (SPR) at the metal-dielectric interface and localized surface plasmon resonance (LSPR) in metallic nanostructures.

### 2.1. Fundamentals of SPR

**Figure 1:**
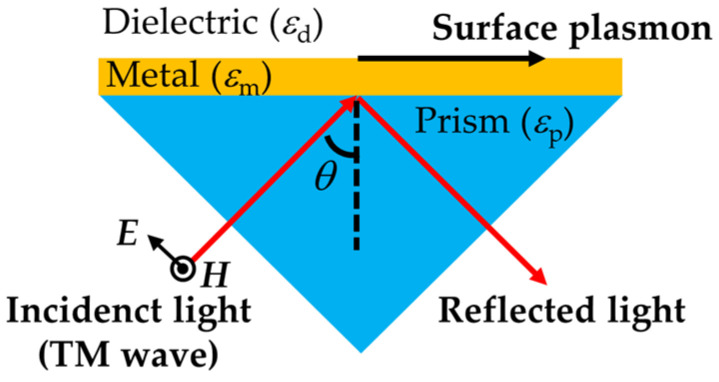
Kretschmann configuration for SPR.

SPR is a traditional plasmonic phenomenon, which is based on the collective oscillation of free electrons in metal film on the interface with a dielectric medium. SPR can be excited in a variety of illumination schemes. Among them, Kretschmann geometry [8] is the most popular configuration by simply depositing a metal film, e.g., a gold layer, on a prism that is used to achieve momentum (wave vector) matching between the incident light and the plasmon (Figure 1). An incident beam with a transverse magnetic (TM) polarization state (the electric field vector is parallel to the incident plane) is illuminated onto the planar dielectric-metal interface from the prism side. When the incident angle *θ* is larger than the critical angle, total internal reflection occurs at the interface, and a part of the light propagates in the metal surface in the form of an evanescent wave. The wave vector of the evanescent wave is equal to the vector component of the incident light (*k*_0_) parallel to the dielectric-metal interface *k*_‖_:

(1) $$k_{∥}=k_{0}\sin\theta =\frac{\omega }{c}\sqrt{\varepsilon_{p}}\sin\theta =\frac{2\pi }{\lambda }\sqrt{\varepsilon_{p}}\sin\theta$$

where *ω* and *c* are the angular frequency of the incident light and the velocity of light in a vacuum, respectively. *λ* is the light wavelength, and *ε*_p_ is the dielectric constants of the prism. At a certain incident angle *θ*_0_, the momentum-matching condition between the evanescent wave and the surface plasmon is fulfilled, causing the surface plasmon wave to be excited through the coupling of oscillating free electrons with the photons of the incident light. As a result, there is a minimum of angle-dependent reflectance, which is the SPR phenomenon.

The crucial properties of surface plasmon can be derived from Maxwell’s equations applied to a flat and infinite metal-dielectric interface. Basically, the dispersion relation of surface plasmon is given by:

(2) $$k_{sp}=\frac{2\pi }{\lambda }\sqrt{\frac{\varepsilon_{m}\varepsilon_{d}}{\varepsilon_{m}+\varepsilon_{d}}}$$

where *k*_sp_ is the wave vector of surface plasmon, *ε*_m_ and *ε*_d_ are the dielectric constants of the metal layer and dielectric medium, respectively. Combined Equation (2) with Equation (1), the resonance condition of SPR can be obtained as follows:

(3) $$\frac{2\pi }{\lambda }\sqrt{\varepsilon_{p}}\sin\theta_{0}=\frac{2\pi }{\lambda }\sqrt{\frac{\varepsilon_{m}\varepsilon_{d}}{\varepsilon_{m}+\varepsilon_{d}}}$$

**Figure 2:**
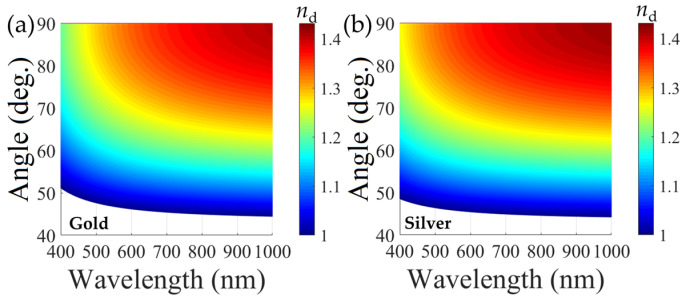
Resonance condition of SPR on an interface of dielectric and (**a**) gold, (**b**) silver with a SiO_2_ prism used.

According to Equation (3), one can obtain insight into the relationship of the incident light wavelength, incident angle, and dielectric constant with given metal and prism materials used. Figure 2 presents the resonance condition of SPR on a metal-dielectric interface through coupling with a SiO_2_ prism (*ε*_p_ = *n*_d_^2^ = 1.45^2^, where *n*_d_ is the refractive index of the dielectric medium in contact with the metal layer). It can be seen that with a given refractive index of the dielectric medium, the incident angle must be larger than a critical value to excite SPR within a certain wavelength range. For instance, when the metal is gold, and the dielectric medium is air (*ε*_d_ = 1), the required incident angle decreases as the wavelength of the incident light increases from 400 to 1000 nm, and the minimum angle is 44.4°. (Figure 2a). Moreover, when the dielectric medium is water (*ε*_d_ = 1.333^2^), the incident angle needs to be larger than 70.8°. In addition, the operation wavelength (incident angle) undergoes a redshift as the refractive index of the dielectric medium increases at a fixed incident angle (operation wavelength). These dependences lay the foundations of plasmonic sensing, including angular modulation and wavelength modulation [6,9,10]. Similar to the behavior of SPR at the gold-dielectric interface, SPR at the silver-dielectric interface requires a critically minimal angle of incidence of 44.2° and 69.6° in air and water, respectively. Note that for a given dielectric constant, the LSPR wavelength/angle is shorter/smaller for silver than that for gold.

As Equation (2) describes, the momentum of the surface plasmon is larger than that of the incident light in free space, causing a momentum mismatch between the light and the surface plasmon. This is the reason why prism couplers are necessary for the excitation of surface plasmon, as mentioned above. Meanwhile, a critically large angle of incidence is needed. Since the guided beam within an optical fiber core is basically paraxial, i.e., the output beam of an optical fiber is nearly normal to the fiber end-facet, the traditional SPR is not able to be excited on common fiber tips [11].

### 2.2. Fundamentals of LSPR

**Figure 3:**
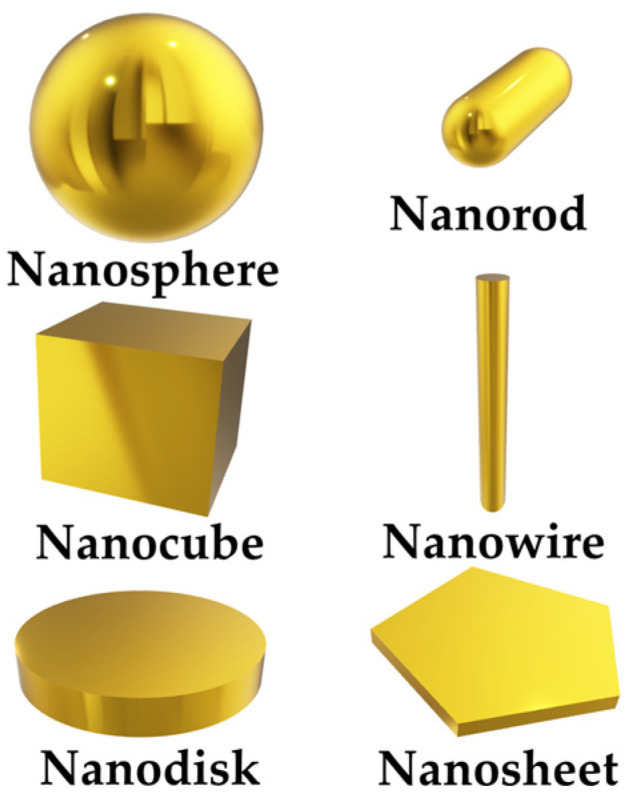
Schematics of configurations of metallic nanoparticles for LSPR.

LSPR is similar to SPR when the surface plasmon is localized [12]. Curved surfaces of bounded geometries (e.g., metallic nanoparticles) with a size comparable to the wavelength of light offer an effective restoring force driving the free electrons to oscillate collectively, resulting in local plasmon resonance. In addition to metallic nano-spheres, voids of various nanoparticle (NPs) topologies support LSPR, such as nanodisks, nanowires, and nanosheets [13], etc., as illustrated in Figure 3.

**Figure 4:**
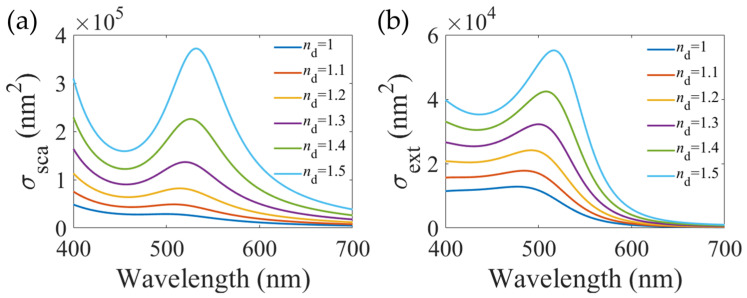
LSPR responses of gold NPs with radius of 50 nm towards the refractive index of surrounding medium: (**a**) scattering and (**b**) extinction cross-section.

Again, the properties of LSPR in NPs can be understood by solving Maxwell’s equations. Mie’s theory is successful in quantitating the scattering and absorption of light by spherical particles [14]. With the sizes of particles appreciably smaller than the light wavelength (quasistatic approximation), applying Mie’s theory to the interaction of metallic NPs and light, the following total scattering, extinction, and absorption cross-sections can be obtained [15]:

(4) $$\sigma_{sca}=\frac{32\pi^{4}\varepsilon_{d}^{2}V^{2}}{\lambda^{4}}\frac{{\left(\varepsilon_{r}-\varepsilon_{d}\right)}^{2}+\varepsilon_{i}^{2}}{{\left(\varepsilon_{r}+\chi \varepsilon_{d}\right)}^{2}+\varepsilon_{i}^{2}}$$

(5) $$\sigma_{ext}=\frac{18\pi \varepsilon_{d}^{3/2}V}{\lambda }\frac{\varepsilon_{i}}{{\left(\varepsilon_{r}+\chi \varepsilon_{d}\right)}^{2}+\varepsilon_{i}^{2}}$$

(6) $$\sigma_{abs}=\sigma_{ext}-\sigma_{sca}$$

where *V* is the volume of the metallic NPs, *ε*_r_ and *ε*_i_ are the real and imaginary parts of the dielectric constants of the metallic NPs, respectively. *χ* represents the geometric factor for metallic NPs (*χ* = 2 for a sphere and up to 20 for a high-aspect ratio particle) [16]. According to Equations (4)–(6), the resonance position of LSPR strongly depends on the dielectric constant of the surrounding medium (Figure 4), offering opportunities for many applications, ranging from detections of external stimuli to light modulations via external stimuli [17,18].

### 2.3. Fundamentals of Periodic Array of Nanofeatures

**Figure 5:**
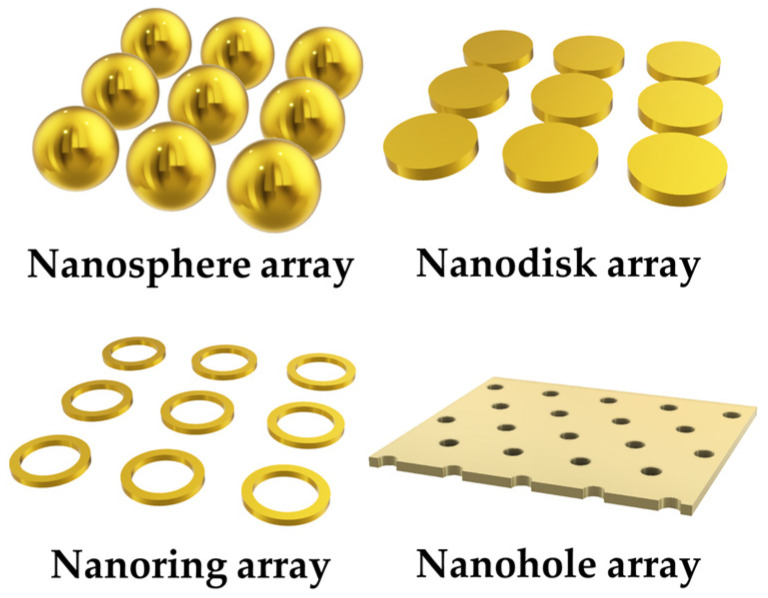
Schematics of configurations of metallic nano-arrays for LSPR.

Beyond the LSPR in isolated metallic NPs, another classic type of nanoplasmonic scheme is the periodic array of nanofeatures (Figure 5) [19]. The electron oscillations are consistent in a periodic array of metallic nanostructures, beneficial to the optimization of resonance intensity and quality factor of the LSPR mode [10,20].

Among the various kinds of plasmonic nano-arrays, nanohole arrays have so far been one of the most intensively investigated and utilized structures [21,22]. In 1998, Ebbessen et al. reported an extraordinary optical transmission (EOT) phenomenon in two-dimensional arrays of subwavelength apertures where the transmission efficiency can exceed unity normalized to the area of the apertures [23,24]. LSPR effect is attributed to be responsible for the EOT. Specifically, a surface plasmon is excited on the metallic surface at the incident side; then, the energy is coupled towards the transmitted side through a tunneling effect followed by the re-emission of surface plasmon to free-space light. The two-dimensional arrangement of nanoholes works as a coupling grating from the incident photon to the surface plasmon as described by:

(7) $$k_{sp}=k_{∥}\pm iG_{x}\pm jG_{y}$$

where ***G***_x_ and ***G***_y_ are the reciprocal lattice vectors for an array assuming the nanohole array is placed on x-y plane. *i* and *j* are integers representing the diffraction orders. The scalar format of Equation (7) is:

(8) $$k_{sp}=\sqrt{{\left(k_{0}\sin\theta \pm iG_{x}\right)}^{2}\pm {\left(jG_{y}\right)}^{2}}$$

For a square array with a lattice pitch of *P*, the reciprocal lattice vectors are *G*_x_ = *G*_y_ = 2π/*P*; for a hexagonal array with the lattice pitch of *P*, the reciprocal lattice vectors are $G_{x}=G_{y}=4\pi /\left(\sqrt{3}P\right)$. Thus, the resonance wavelengths *λ*_r_ of EOT associated with square and hexagonal arrays of metallic nanoholes in a first approximation (that the plasma dispersion of the films remains unchanged) are given by:

(9) $$\lambda_{r}≅\frac{P}{\sqrt{i^{2}+j^{2}}}\left(\sqrt{\frac{\varepsilon_{m}\varepsilon_{d}}{\varepsilon_{m}+\varepsilon_{d}}}\pm \sin\theta \right)$$

and

(10) $$\lambda_{r}≅\frac{P}{\sqrt{\frac{4}{3}\left(i^{2}+ij+j^{2}\right)}}\left(\sqrt{\frac{\varepsilon_{m}\varepsilon_{d}}{\varepsilon_{m}+\varepsilon_{d}}}\pm \sin\theta \right)$$

respectively. Considering the fact that the absolute value of the dielectric constant of noble metals is normally much larger than that of the surrounding media (|*ε*_m_|»*ε*_d_), Equations (9) and (10) can be simplified into:

(11) $$\lambda_{r}≅\frac{P}{\sqrt{i^{2}+j^{2}}}\left(\sqrt{\varepsilon_{d}}\pm \sin\theta \right)=\frac{P}{\sqrt{i^{2}+j^{2}}}\left(n_{d}\pm \sin\theta \right)$$

and

(12) $$\lambda_{r}≅\frac{P}{\sqrt{\frac{4}{3}\left(i^{2}+ij+j^{2}\right)}}\left(\sqrt{\varepsilon_{d}}\pm \sin\theta \right)=\frac{P}{\sqrt{\frac{4}{3}\left(i^{2}+ij+j^{2}\right)}}\left(n_{d}\pm \sin\theta \right)$$

respectively. According to Equations (11) and (12), the resonance wavelength of the nanohole arrays depends on the geometrical parameters of the lattice and the dielectric properties of the surrounding medium, i.e., scaling with the lattice pitch of the nano-array and/or the refractive index of the surrounding medium. This characteristic opens up the way to tune the properties of metallic nanohole arrays and detect external variations in dielectric conditions. It is found that the sensitivity of nanohole arrays towards external changes in refractive index is proportional to the lattice pitch [25]. However, there is a tradeoff between sensitivity and local field enhancement. A smaller pitch (closer nanohole spacing) benefits the coupling between adjacent nanofeatures leading to a stronger electric field enhancement locally known as “hot-spot” [26]. However, as the nanofeatures become closer, the resonance peak may broaden due to the coupling and overlapping of LSPR mode around the nanofeatures. As a result, the EOT spectrum evaluates into a wide resonant peak, limiting the quality factor of the related nanostructures [6].

According to the above discussions, the essential difference between SPR and LSPR is that the LSPR can be excited by direct light illumination, i.e., the formation of LSPR on the surface of metallic nanostructures does not need to meet the momentum matching condition [27]. Considering the near-normal illumination condition in optical fiber tips, metallic nanostructures with LSPR responses are favorable to constructing plasmonic fiber tips.

## 3. Plasmonic Functionality of Optical Fiber Tips

Fabrication approaches of plasmonic nanostructures involve bottom-up and top-down procedures [28,29]. Bottom-up methods rely on the synthesis of materials and chemical reactions to grow plasmonic nanostructures in dimension. Bottom-up fabrications are attractive due to the low cost and mass production advantages, while the sacrifice of precise control of the geometrical physical parameters of the nanostructures hinders advanced applications. On the hand, top-down methods directly pattern nanostructures onto metallic substrates with controlled shapes and sizes by removing materials selectively. Nevertheless, most top-down fabrication methods suffer from high cost and low efficiency.

The flat tip of an optical fiber facilitates the popular nanofabrication techniques to functionalize optical fiber tips for plasmonic applications [4], but proper optimizations of the fabrication procedures are required. For example, optical fibers have small diameters, large aspect ratios, and fragile bodies, making the preparation and handling difficult in plasmonic functionalities. In recent years, transfer methods for plasmonic functionalities of optical fiber tips are emerging [30]. In transfer methods, desired plasmonic nanostructures are firstly fabricated on original substrates and then transferred onto fiber tips flexibly and efficiently [31].

### 3.1. Bottom-Up Methods

#### 3.1.1. Self-Assembly

Self-assembly (SA) is the simplest approach to functionalizing the pristine fiber tips [32]. Self-assembly enables convenient and high throughput productions of plasmonic fiber tips with attached NPs via the natural noncovalent bonds (e.g., electrostatic attraction). The earliest report of using the SA method to functionalize an optical fiber tip was demonstrated in 1995 [33]. Stokes et al. dipped a polished optical fiber tip into a 5% aqueous suspension of alumina for about three seconds, ensuring adequate attaching of NPs. The silver deposition was then performed in a vacuum evaporation system to coat a 100 nm thick silver layer on top of the alumina NPs on fiber tips. The silver-coated alumina microsphere-attached fiber tips were of plasmonic functionality. They claimed the potential of the small tip size design for micro-scale sensors of in situ measurements. In 2000, the same group optimized the fiber tip structure and demonstrated the application in surface-enhanced Raman scattering (SERS) [34]. In the same year, Viets et al. found that the performance of SA-based plasmonic fiber tip could be enhanced by a factor of 6 through angled polishing of the pristine tip [35]. The intensity increased monotonically with the increase in the angle and reached its maximum value at the fiber-polished angle of 40°. In 2003, Cheng et al. functionalized gold NPs on fiber tips with glycine, succinic acid, or biotin to enhance the selectivity of the sensor [36]. Adapting the SA method, Shi et al. demonstrated a new design of a “sandwich” plasmonic functionality structure on fiber tips in 2008 [37]. Firstly, the fiber tip was coated with silver NPs via the SA method. Then, the plasmonic fiber tip was dipped into a solution with a mixture of silver NPs and target analyte molecules to form a random silver nanoparticle-analyte molecule sandwich structure. It was found that the sensitivity was significantly improved, benefitting from the extremely large electric field enhancement in the gap of adjacent silver NPs. In 2010, Andrade et al. presented a single-mode fiber tip with a core radius of 2 μm by self-assembling silver NPs onto the fiber tip using 3-aminopropyltrimethoxysilane [38]. Furthermore, they optimized the NPs-modified optical fiber tips through a “layer-by-layer” procedure. It was shown that the performance was optimal when 5 “layers” of 50 nm silver-NPs were deposited on the optical fiber tip. They evaluated the stability of the plasmonic fiber tip as well. It was found that there was no performance loss for the fiber tip when it was stored for 3 days in the air. Nevertheless, the fiber tip degrades dramatically after storage for 24 h in water and completely turns invalid after storage in a methanolic solution for the same period of time. They attributed the degradation of the device performances to the solvolysis of the silane linker layer contact with water and methanolic medium [39].

**Figure 6:**
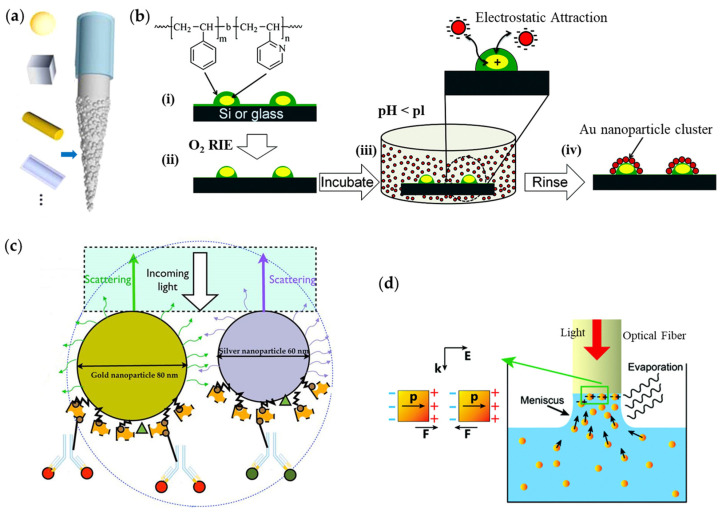
Self-assembly methods for fabrications of plasmonic fiber tips. (**a**) Shapes of self-assembled metallic NPs. Reproduced with permission [40]. Copyright 2015, American Chemical Society. (**b**) Template-guided SA approach. Reproduced with permission [41]. Copyright 2012, American Chemical Society. (**c**) Multiplexed fiber tip via SA method. Reproduced with permission [42]. Copyright 2014, American Chemical Society. (**d**) Laser-induced SA method. Reproduced with permission [43]. Copyright 2016, The Royal Society of Chemistry.

Note that the SA method is efficient in functionalizing optical fiber tips with varied shapes of NPs for plasmonic aims (Figure 6a) [40]. However, the plasmonic nanostructures produced by SA are basically arranged in a random way and are hard to be controlled on demand. In order to address this issue, attempts have been made in very recent years. Yap et al. tried to arrange gold nanoparticle clusters onto optical fiber end-facets with a copolymer templated-guided SA method [41], as shown in Figure 6b. Gold nanoparticle cluster arrays were fabricated by SA of gold NPs guided by nanopatterns of poly (styrene-block- 2-vinylpyridine) reverse micelles on silicon or glass substrates. Cluster formation is driven by electrostatic self-assembly of anionic citrate-stabilized gold NPs (∼11.6 nm diameter) onto two-dimensionally ordered polyelectrolyte templates. The two-dimensional quasi-hexagonally periodic array of the polymer template could be tuned by adjusting the spin-coating speed. They have successfully demonstrated the tunability of nanoparticle numbers within one cluster (5, 8, 13, or 18) as well as the spacing of clusters (37–10 nm). This method worked well on planar substrates, while disorders of clusters appeared for optical fiber tips. They suggested possible optimizations of the used fibers and employed processing could be helpful in overcoming this problem. In 2014, Sciacca et al. reported a multiplexed biosensor with one single fiber tip by attaching both gold and silver NPs onto the fiber tip (Figure 6c) [42]. Due to the distinct LSPR positions from gold and silver NPs, the fiber tip could detect different analytes simultaneously with a limited cross-reactivity and in a short time frame. It was found that the deposited nanoparticle density had a significant impact on the sensor performance, i.e., smaller quantities of NPs led to better sensitivity. It was also suggested that the using of core-shell NPs, along with the tunability of operation wavelength, is helpful for improving the sensor performance furthermore. Liu et al. proposed a laser-induced SA method to fabricate a plasmonic fiber tip with high sensitivity and excellent reproducibility (Figure 6d) [43]. A meniscus forms around the fiber tip due to the surface tension of the suspension liquid that contains pre-synthesized NPs as the fiber facet is above the colloid surface. Then guided laser within the fiber would induce thermal effects on the NPs in the meniscus that controlled the deposition of NPs on fiber tips, and the electromagnetic interactions among the closely spaced NPs help this arrangement.

#### 3.1.2. Nanosphere Lithography

**Figure 7:**
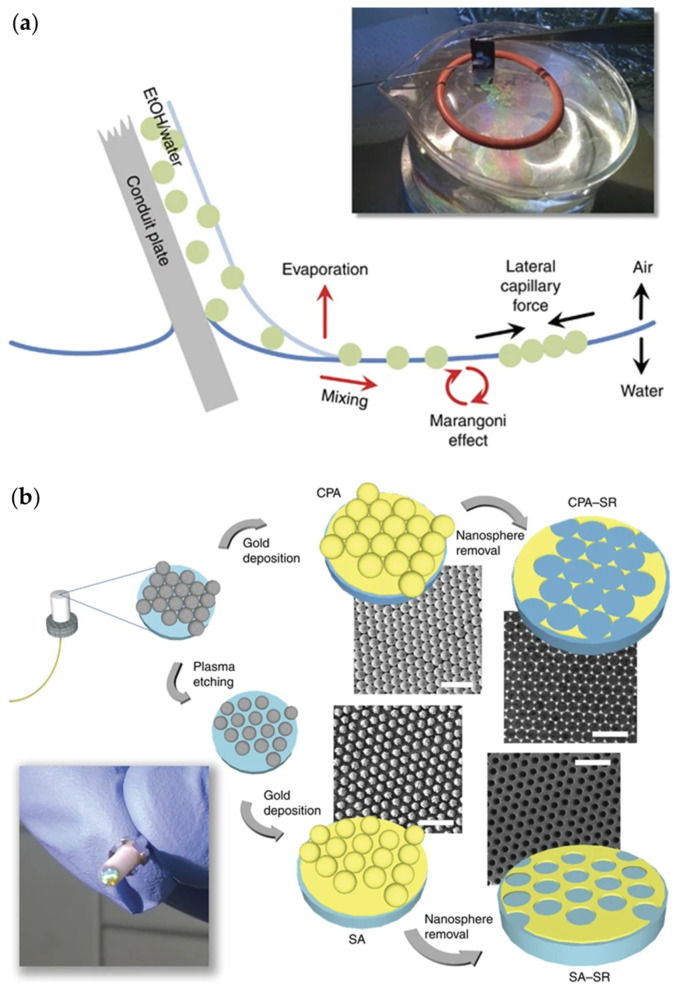
Nanosphere lithography for plasmonic functionalities of optical fiber tips [47]. (**a**) Schematic of the nanosphere assembly process, (**b**) schematic procedure of the fabrication of different fiber tips and SEM images (scale bar = 3 μm) corresponding to each geometric feature: close-packed array (CPA), sparse array (SA), and sphere removal (SR).

Nanosphere lithography (NSL) is a bottom-up nanofabrication technique based on the advanced SA method, which is capable of building well-ordered two-dimensional nano-array structures [44,45]. In the NSL procedure, monodisperse nanospheres are self-assembled in a hexagonal array on the pristine substrate first. Then, the nanosphere array is transferred onto a target substrate, e.g., optical fiber end-facet, to work as the nanostructure template. After metallic layers are deposited, forming plasmonic elements, the assembled nanospheres are dissolved by a proper solvent, leaving the desired plasmonic pattern on the fiber tip. For instance, Pisco et al. adapted the so-called breath figure methodology to functionalize nonconventional substrates, such as optical fibers, enabling the direct formation of regular and ordered metal-dielectric nanostructures on fiber tips for plasmonic purposes [46,47]. As shown in Figure 7, during the fabrication, a polymeric honeycomb was first deposited on fiber end-facet. Due to the evaporation of the polymer solution, water droplets were auto-organized into a hexagonal pattern at the liquid/air interface (Figure 7a). After the complete evaporation of the solution, a hexagonal imprint as a template was produced on the polymer, followed by the deposition of desired metallic layers. The method is efficient and powerful in constructing several configurations with varied geometrical features on fiber tips (Figure 7b). The successful functionalization was confirmed by the corresponding scanning electron microscope (SEM) images.

### 3.2. Top-Down Methods

#### 3.2.1. Focused-Ion Beam Milling

**Figure 8:**
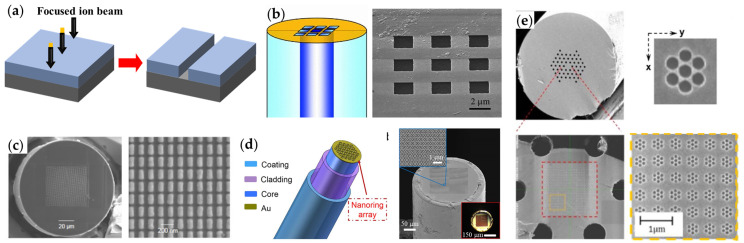
FIB milling for plasmonic functionalities of optical fiber tips. (**a**) Schematic of FIB milling process. (**b**) Nano-aperture array on single-mode fiber tip. Reproduced with permission [48]. Copyright 2006, IOP Publishing Ltd. (**c**) Nanorod array on multi-mode fiber tip [49]. (**d**) Nanoring array on multi-mode fiber tip. Reproduced with permission [50]. Copyright 2017, American Chemical Society. (**e**) Heptamer-arranged nanoholes on PCF tip. Reproduced with permission [51]. Copyright 2021, Optical Society of America.

Focused-ion beam (FIB) milling is an advanced nanofabrication technique that impinges a focused beam of ions (typically Ga) onto the target substrate to ablate surface materials to directly pattern nanostructures (Figure 8a). Extensive efforts have been made to functionalize optical fiber tips with the FIB method. The first work on FIB-milled plasmonic fiber tips was reported in 2006 [48]. Nellen et al. investigated the direct writing of micro-structures on optical fiber tips with FIB. Cleaved tips of optical fibers were coated with a gold film with a thickness of 50 nm using the sputtering method. As shown in Figure 8b, the gold film within the core region of the fiber was patterned to form various square apertures. They demonstrated the transmission function of the apertured fiber tip by the diffraction pattern measured in the Fraunhofer regime. Later, Smythe et al. presented a systematic investigation of optical antenna arrays and used FIB to fabricate a plasmonic optical antenna fiber probe [49]. A 3 nm sticking layer of titanium and 35 nm of gold were first deposited on the end-facet of a multi-mode fiber, and then FIB was used to selectively remove the gold and titanium, leaving behind arrays of gold nanorods (Figure 8c). The fiber tip offers a plasmonic functionality and can potentially be used for in situ chemical and biological detection and SERS. However, plasmonic nanostructures suffer from the broad linewidth and low peak-to-dip signal ratio in the extinction spectra due to the strong radiative damping. In 2017, Liang et al. fabricated a plasmonic nanoring resonator array onto an optical fiber tip using FIB milling (Figure 8d) [50]. Benefitting from the strong coupling of the dipolar modes from the complex nanohole–nanodisk structures, the subradiant lattice plasmon resonance supports narrow linewidth with a high peak-to-dip signal ratio and strong near-field electromagnetic enhancement. This plasmonic fiber tip is ideal for high-sensitivity chemical and biomedical sensing. Recently, Suleman et al. demonstrated a plasmonic photonic crystal fiber (PCF) tip using FIB milling [51]. The FIB method was used to fabricate resonant plasmonic nanostructures onto the end-facet of a solid-core polarization-maintaining PCF. The implemented nanostructures consist of an array of heptamer-arranged gold nanoholes covering the solid core of the PCF. The nanoholes were milled with a focused He^+^ beam, as shown in Figure 8e.

In general, the FIB technique allows high-resolution patterning and requires no mask on materials ranging from glass and fiber to metals. Nevertheless, FIB suffers from some critical disadvantages. For instance, the contamination (ion doping) of the sample substrate is unavoidable because of the poor conductivity of optical fibers, though the issue is balanced by pre-depositing metallic films to a certain extent. In addition, angled sidewalls of the patterned features are common during milling, which requires extra optimizations in the design details and fabrication processes. Moreover, FIB milling is generally time-consuming, and large-area nanostructures are almost impossible to be processed. Thus, FIB milling is limited to proof-of-concept applications of plasmonic fiber tips.

#### 3.2.2. Electron-Beam Lithography

**Figure 9:**
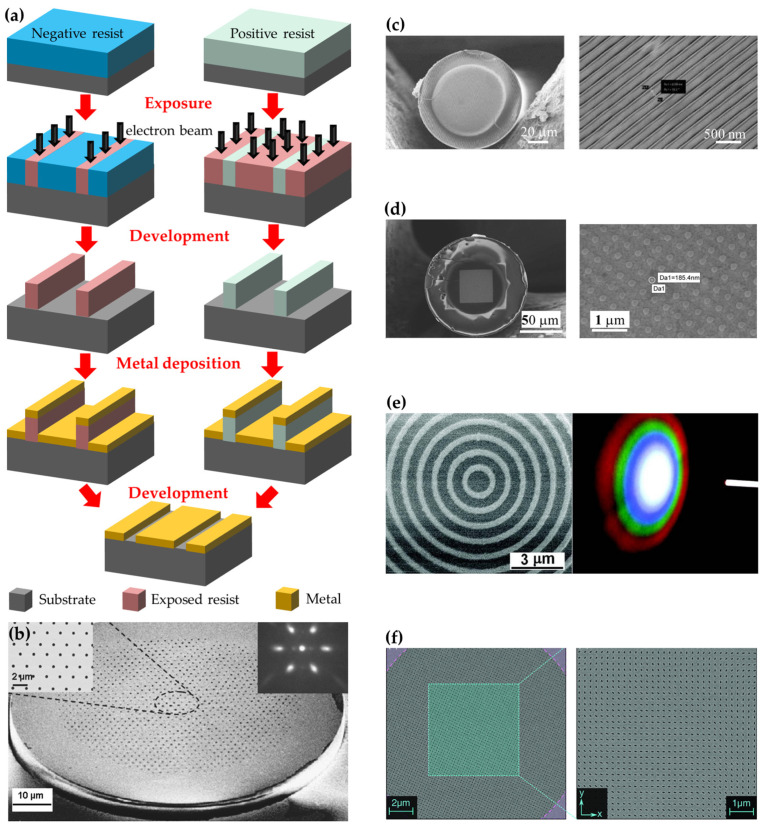
EBL patterning for plasmonic functionalities of optical fiber tips. (**a**) Schematic of EBL process, (**b**) nanohole array. Reproduced with permission [52]. Copyright 2007, American Institute of Physics. (**c**) Nano-grid [53]. (**d**) Nanodot array on single-mode fiber tip [54]. (**e**) Concentric gold ring grating on multi-mode fiber tip. Reproduced with permission [55]. Copyright 2012, Wiley-VCH Verlag GmbH & Co. KGaA, Weinheim. (**f**) Metasurface on single-mode fiber tip [56].

Similar to FIB, electron-beam lithography (EBL) utilizes a focused electron beam to produce micro- and nanostructures with arbitrary shapes in a two-dimensional plane. As lithography, electron irradiation induces a chemical modification of the electron beam to resist, i.e., the solubility of the resist is altered. By immersing the resist in a solvent, either the exposed (positive resist) or non-exposed (negative resist) parts of the resist can be selectively removed, as schematically shown in Figure 9a. The resist patterns produced by EBL serve as masks for subsequent deposition or etching on fiber tips. In the last two decades, EBL has witnessed success in the fabrication of plasmonic fiber tips thanks to its versatility and ultimate resolution (smaller than 10 nm). The first work on plasmonic fiber tip completed by EBL was reported in 2007. Tian et al. used EBL to produce a nanohole array in metallic coatings on cleaved single-mode optical fibers, where the diameter of the nanohole ranges from 100 to 700 nm, and the pitch of the array is varied from 300 to 3000 nm (Figure 9b) [52]. Both square and hexagonal arrays are feasible to be produced on the fiber tips using EBL. Light transmissions at 632.8 and 1550 nm showed the expected effects of nanohole size, spacing, array symmetry, and wavelength. Later on, Lin et al. proposed an optical fiber inline polarizer achieved by EBL-fabricated metallic nano-grid on a single-mode fiber tip at the wavelength of 1550 and 1310 nm (Figure 9c) [53] and gold nanodot array with a periodicity of 400 nm, a diameter of 185 nm, and 55 nm in height (Figure 9d) [54]. In 2012, Feng et al. fabricated a concentric gold ring grating with a period of 900 nm on the end-facet of and multi-mode optical fiber with a core diameter of 600 μm using a combination of lift-off process and EBL (Figure 9e) [55]. In detail, a 30 nm thick gold layer was evaporated onto the polished fiber end-facet. This initial gold film helped make the facet surface electrically conducting in order to avoid electric charging during the followed EBL process. In 2012, Zeisberger et al. demonstrated the successful functionalization of a fiber tip with a plasmonic metasurface consisting of gold nanoslots on the end-facet of a modified single-mode fiber via EBL technique (Figure 9f) [56].

According to the above cases, one can easily see that EBL is similar to FIB. EBL technique provides a precise and versatile approach to integrating plasmonic nanostructures on optical fiber tips. Moreover, EBL offers the advantage of large-area patterning at the order of 100 μm × 100 μm, which covers the typical dimensions of optical fiber tips. However, EBL shares some drawbacks of FIB milling. For instance, EBL suffers from angled sidewalls of patterned structures and the time-consuming nature of the patterning process. Additionally, the required lift-off step for metallization hinders the creation of high aspect ratio plasmonic nanostructures.

#### 3.2.3. Nanoimprinting Lithography

**Figure 10:**
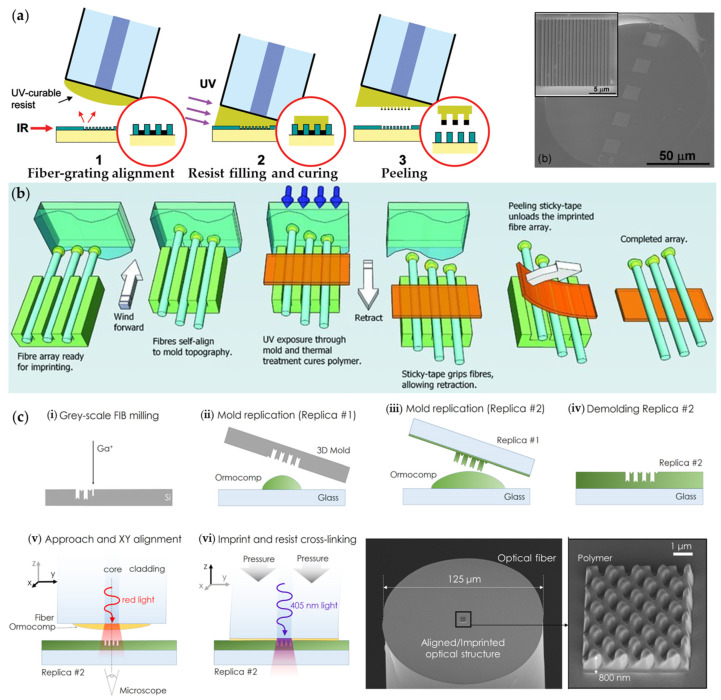
NIL technique for plasmonic functionalities of optical fiber tips. (**a**) Schematic for defining surface relief gratings on the fiber tip and the SEM image of the plasmonic fiber tip. Reproduced with permission [58]. Copyright 2008, American Institute of Physics. (**b**) Schematic of a high-throughput and self-aligned, array-imprinting steps in contact with customized molds. Reproduced with permission [59]. Copyright 2011, WILEY-VCH Verlag GmbH & Co. KGaA, Weinheim. (**c**) Schematic for nanoimprinting of a 3D structure on an optical fiber and the SEM image. Reproduced with permission [60]. Copyright 2016, IOP Publishing Ltd.

Nanoimprinting lithography (NIL) is a mechanical patterning technique that allows the creation of large-area, high-resolution, nanometer-scale functional structures with a nature of high-throughput capacity. Due to the mechanical modification of host substrates in NIL, only soft or thermally softened materials (typically polymers) are available for NIL processing. In addition, the resolution of nanoimprinted features is limited by the applied material properties and the accuracy of the template rather than the electron/ion scattering during FIB/EBL processing. Therefore, for plasmonic functionalities of optical fiber tips with NIL, it starts with coating curable polymer onto fiber tips, followed by feature replications, curing, and metallic layer depositions [57]. In 2008, Scheerlinck et al. used the NIL method to attach gold film with the imprinted polymer to propose strong diffraction gratings at the fiber tip, shown in Figure 10a. The plasmonic fiber tip was employed as a fiber-optic grating coupler between optical fibers and chip waveguides [58]. In order to address the alignment issue of small-sized optical fibers with molds, Kostovski et al. developed a parallel, self-aligned and portable NIL for efficient and high-throughput fabrications of plasmonic fiber tips [59]. As shown in Figure 10b, they utilized a row of U-shaped grooves on the stage to confine the applied fibers and then contacted the polymer-coated fiber tips with the molds (antireflection nanostructure on a cicada wing and self-assembled nanostructure of anodized aluminum oxide) to form the imprinted features. Silver layers were deposited onto the cured polymer nanostructures to construct SERS probes. NIL is capable of producing complex (e.g., three-dimensional) structures on optical fiber end-facet as well. In 2016, Calafiore et al. demonstrated the fabrication of three-dimensional structures for light wavefront manipulation on the end-facet of an optical fiber by NIL [60]. Firstly, multilevel features were obtained by grayscale gallium-FIB milling on a flat substrate (silicon with minimal charging), and then, the final nanofeatures on fiber tips were formed by a sequence of two replications, as schematically shown in Figure 10c.

### 3.3. Transfer Methods

The aforementioned nanofabrication methods are all based on patterning nanostructures directly on optical fiber tips. Though significant success has been witnessed in the direct plasmonic-functionalization of optical fiber tips, the fabrication resolution (performances) and efficiency (cost) often conflict with each other. In the last years, transfer methods as an alternative technique to conventional nanofabrication schemes have been developed in functionalizing optical fiber tips with plasmonic elements. Basically, the transfer methods are based on transferring pre-fabricated nanostructures from planar substrates to fiber end-facets.

**Figure 11:**
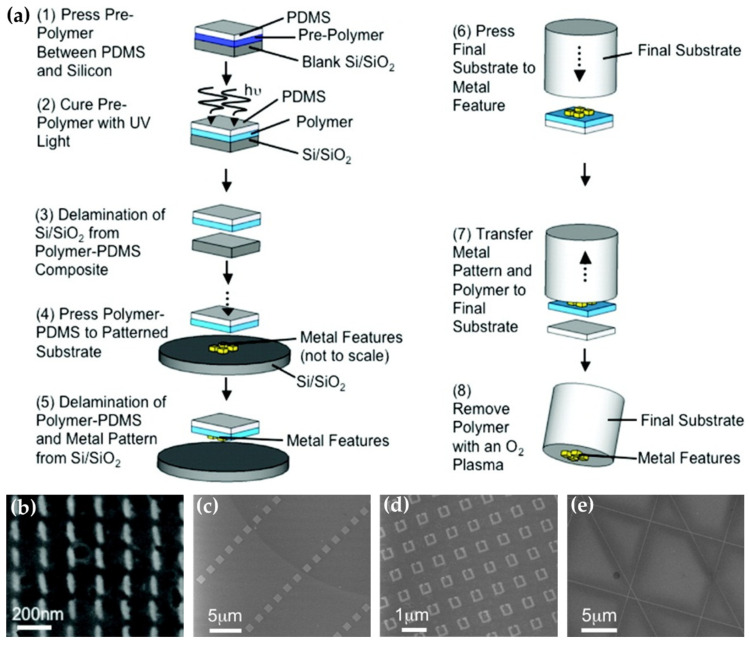
First demonstrations of transfer method for fabrications of plasmonic fiber tips. (**a**) Schematic process of the transfer method, (**b**–**e**) SEM images of transferred nanorods, nanocubes, nano split rings, and nanowires on fiber end-facets. Reproduced with permission [61]. Copyright 2009, American Chemical Society.

The very initial demonstrations of the transfer concept were reported by Smythe et al. in 2009 [61,62]. Firstly, gold nanostructures were produced on Si/SiO_2_ substrate (silicon with a native oxide layer) using EBL. Then, a sacrificial polymer (thiol-ene) film bearing thiol groups was used to strip the metallic features from the Si/SiO_2_ substrate. The thiol-ene film, bearing the nanofeatures, was gently pressed down to contact the target substrate surface. At last, the sacrificial thiol-ene film was removed with an oxygen plasma, leaving the pattern of gold nanostructures attached to the final target substrate, e.g., optical fiber end-facet (Figure 11). Metallic nanofeatures, including nanorods, nanocubes, nanowires, nano split rings, and nanodots ranging from 30 nm to 1 μm in size, had been successfully transferred onto optical fiber end-facet without disturbing the distributions of the nanofeatures. Once the versatility of the transfer technique has been proved, it becomes one of the favorite methods for constructing plasmonic fiber tips.

**Figure 12:**
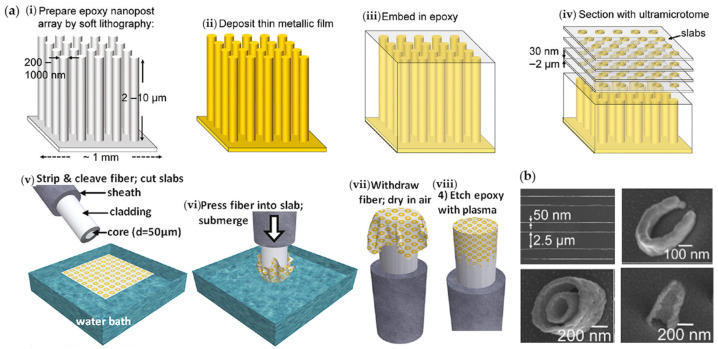
Nanoskiving transfer method for plasmonic functionalization of optical fiber tips. (**a**) Schematic process, (**b**) SEM images of transferred nanofeatures. Reproduced with permission [63]. Copyright 2010, American Chemical Society.

The above transfer method is only appropriate for those materials that do not form covalent bonds with the substrate, allowing the material to be stripped to complete the transfer. In order to overcome the limitation, the same group conceived a simplified transfer process using the nanoskiving and wet-transfer method [63], shown in Figure 12. In the nanoskiving process, the epoxy nanopost array prepared by soft lithography was coated with thin metallic films, and then embedded into the epoxy matrix and finally sectioned into slabs by an ultramicrotome. The slabs floated on the water surface and were transferred onto the fiber end-facet by submerging the fiber tip. At last, the carrier film was etched with air plasma, leaving free-standing nanofeatures on the fiber tip.

**Figure 13:**
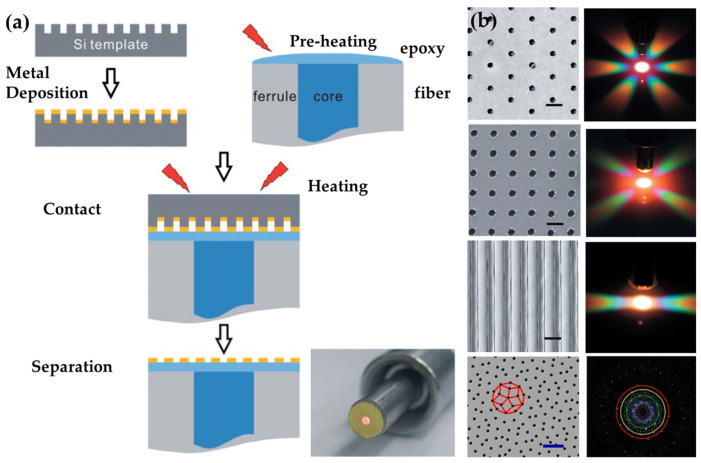
Template and transfer method for plasmonic functionalization of optical fiber tips. (**a**) Schematic process. Reproduced with permission [30]. Copyright 2014, The Royal Society of Chemistry 2014. (**b**) SEM images and far-field diffractions of transferred nanohole array, nanoslit array, and quasi-periodic nanohole array. Reproduced with permission [30]. Copyright 2014, The Royal Society of Chemistry. Reproduced with permission [66]. Copyright 2016, American Chemical Society.

Indeed, the transfer of nanofeatures onto fiber tips can be performed without the need for a carrier film, launching the “contact and separate” approach. Firstly, the optical fiber end-facets are coated with an adhesive layer such as curable resist and put in contact with the planar substrates supporting the pre-prepared metallic nanostructures. After the complete curing of the adhesive layer, the nanostructures are separated from the original substrate and strongly bonded onto the fiber end-facets [64]. Since 2013, Jia et al. have achieved the transfers of plasmonic nano-arrays from metallic-coated silicon templates onto optical fiber end-facets by attaching the nanostructures with an adhesive layer covering the fiber end-facets [30,65,66]. The transferred gold nanostructures included a nanohole array, nanoslit array, quasi-periodic nanohole array, etc. (Figure 13).

**Figure 14:**
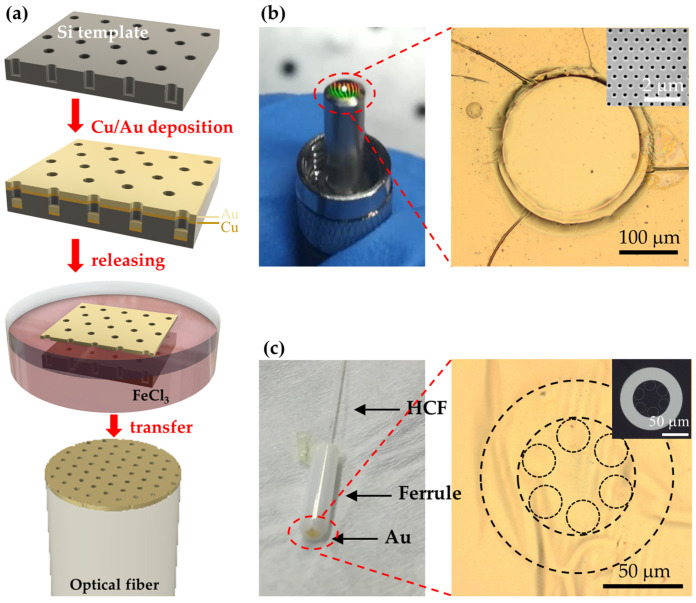
SA-combined template-transfer method for plasmonic functionalization of optical fiber tips. (**a**) Schematic process. (**b**) Multi-mode fiber tip. Reproduced with permission [67]. Copyright 2020, Optical Society of America. (**c**) Hollow-core fiber tip [68].

On the same line of argument, our group further simplified the template transfer method by introducing the SA as the alternative integration way of metallic nanofeatures to adhesive attaching [67,68,69]. As shown in Figure 14, large-area and highly uniform gold nanomembranes with nanohole array were fabricated on silicon wafers by EBL patterning and subsequent metal depositions (copper layer as the sacrificial medium and gold layer as the functional medium). The silicon substrate was used as the template for recyclable transfers. During the transfer process, a wet etching method was applied to release the gold nanomembranes off the template, and the freestanding nanomembranes were then transferred onto the end-facets of different types of optical fibers, including normal multi-mode fibers and hollow-core fibers (HCF). Sufficient van der Waals force maintains the good bond of the gold nanomembranes. Additionally, rather than transferring the gold nanomembrane by a, relatively, time-consuming thermal or ultraviolet-curable adhesive, the non-adhesive template transfer process is more flexible and efficient.

In general, the above typical techniques are capable of fabricating plasmonic fiber tips with high-resolution and good repeatability. In addition, more developed techniques are proposed as well. For instance, femtosecond (fs) laser ablation [70,71] was found to be faster and cheaper than those based on, for example, FIB milling, and a minimal impact on the surrounding materials is involved. However, the surface of the patterned features is generally rougher. The interference lithography technique enables a large area of well-defined periodic nanofeatures in one, two, three dimensions, or even concentric ring patterns based on maskless exposures of different light beam combinations [72,73]. However, the main drawbacks of this technique include the limited resolution (due to diffraction limitation of light) and the only suitability for array structures [74]. Transfer methods are developed to strive to combine the advantages of advanced nanofabrication techniques, e.g., high resolution, robustness, flexibility, cost-efficient, and high yield. Notably, the main trend of emerging nanofabrication techniques is basically demonstrated by striving to modify beyond the transfer methods [75,76,77].

## 4. Applications

The above section discussed numerous studies that demonstrated the plasmonic functionalization of optical fiber tips. The plasmonic fiber tips take many advantages, such as miniaturization, robustness, multifunctionality, and many others. In addition, the light-matter interaction is bloomed in the plasmonic fiber tips due to the nature of plasmonic modes. Therefore, sensing and detection are the most established applications among plenty of potentials.

### 4.1. Bio-Chemical Sensing

At the very beginning of demonstrations, plasmonic fiber tips had been employed in bio-chemical sensing. For example, the first plasmonic fiber tip produced via the natural SA method was used as a SERS probe [33]. Actually, the simplest sensing application of plasmonic fiber tips is to detect changes in the surrounding refractive index of their environment through shifts of the LSPR peak wavelength. The molecular binding event would modify the surrounding refractive index, therefore, leading to the wavelength shift of the plasmonic fiber tips. Sciacca et al. used the SA-fabricated plasmonic fiber tips for multiplexed biosensing [42]. Both gold and silver NPs were attached to the fiber tips with distinct LSPR signatures, and a sensitivity of 387 nm/RIU (refractive index unit) and biological read of binding events were achieved. Plasmonic fiber tips with nano-arrays fabricated by advanced techniques have been demonstrated in bio-chemical sensing [30,68,78,79].

**Figure 15:**
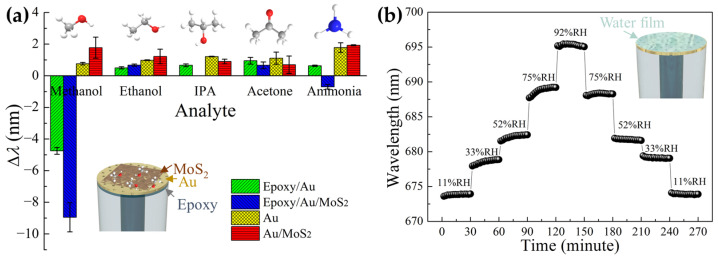
Gaseous sensing of plasmonic fiber tips for (**a**) methanol detection. Reproduced with permission [80]. Copyright 2019, Elsevier. (**b**) Relative humidity detection. Reproduced with permission [67]. Copyright 2020, Optical Society of America.

In addition to the bio-chemical sensing in aqueous media, gaseous sensing has been demonstrated as well. For example, in 2020, our group demonstrated a methanol-selective plasmonic fiber tip [80]. An epoxy rich in hydroxyl groups was used as a sensitive polymer material to bond methanol molecules selectively, and a MoS_2_ monolayer coating was applied to improve the sensing response and selectivity further (Figure 15a). Later on, we observed the relative humidity (RH) response of an SA-fabricated plasmonic fiber tip [67]. Fast water condensation/evaporation on the gold surface is responsible for the high performance of the fiber tip in response to RH. A high sensitivity of 279 pm/% RH is obtained in the range of 11~92% RH. In taking advantage of the fast dynamics (response and recovery times of 156 and 277 ms), the plasmonic fiber tip was capable of monitoring diverse respiration patterns (Figure 15b). Similar work was reported by Liu et al. [81], where they modified a transferred plasmonic fiber tip with silk fibroin film, the refractive index of which was sensitive to external RH, enabling the dependent of LSPR wavelength on RH.

Generally, the sensing principle of plasmonic fiber tips is that the dielectric alteration on the metallic nanostructure interfaces, such as refractive index change, temperature variation, or molecule binding, results in the resonant wavelength(s) shifting. According to this principle, with proper sensitive media functionalized on plasmonic fiber tips, various sensing applications, including thermal distinguish [82], ultrasound detection [83,84], radiation dosimeters [85,86], and magnetic field detection [87] were demonstrated, recently.

### 4.2. Light Field Manipulations

Illuminated by the successful integration of plasmonic functionalities with optical fiber tips, new features were brought to conventional optical fibers beyond sensing [4]. To date, light field manipulation is another research hotspot second to sensing based on plasmonic fiber tips.

**Figure 16:**
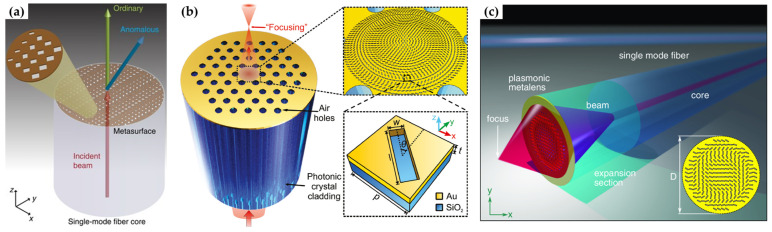
Plasmonic fiber tip integrated with metallic metasurfaces. (**a**) Beam steering with single-mode fiber [89]. (**b**) Focusing with PCF [90], (**c**) focusing with coreless glass [56].

In 2009, Lin et al. demonstrated a fiber in-line polarizer by integrating metallic nano-grid onto fiber end-facet [53]. Plasmonic nano-grating was also used to enable beam generation [88]. In 2017, Principe et al. adapted the concept of metasurface to propose a single-mode optical fiber tip for beam steering [89]. As shown in Figure 16a, a linear-phase distribution covering the 2π range was introduced to enable the transmitted beam to be guided in desired directions. This phase delay was achieved by tuning the side lengths of the gold rectangle nanoholes. Another type of plasmonic fiber tip integrated with metallic metasurface is for beam focusing. In 2019, Yang et al. proposed an on-fiber lens by patterning the core area of a photonic crystal fiber (PCF) end-facet with a circular gold metasurface using FIB milling [90]. As shown in Figure 16b, a gold rectangle nanohole was used as the unit element, and the orientation angle was varied radially to provide the required phase profile for beam focusing. Experimentally, in-fiber meta-lenses with focal lengths of 28 μm and 40 μm at a telecom band of 1550 nm were demonstrated with maximum enhanced optical intensity as large as 234%. Recently, Zeisberger et al. demonstrated a high-numerical-aperture (NA) lens based on a plasmonic fiber tip functionalized with gold nanoslit metasurface [56]. The key feature of the fiber tip is the combination of a coreless glass with gold metasurface, allowing a large NA of 0.3 after the illumination beam expanded up to 48 μm by the coreless glass (Figure 16c).

In addition to the above light field manipulation applications of plasmonic fiber tips, extended cases were proved, including beam coupling [58], filter [91], beam splitter [60], imaging [92], and many others.

### 4.3. Nonlinearity and Lasing

**Figure 17:**
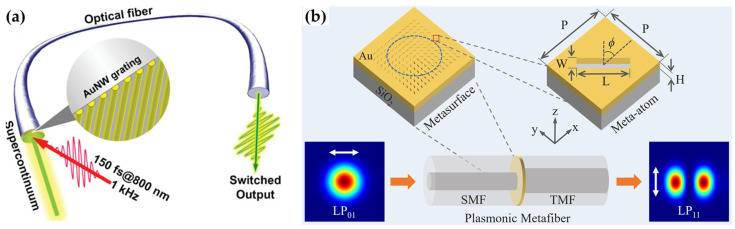
Schematic of plasmonic fiber tips for (**a**) optical switching [74], (**b**) vector lasers [95].

Most recently, plasmonic fiber tips have been used in nonlinearity and lasing. For example, in 2021, Yang et al. achieved the fiber tip-based optical switching [74]. Interference lithography was employed to produce gold nanograting on fiber end-facet. Interaction between the LSPR of the gold nanograting and the band-edge plasmon induced by femtosecond laser pulse excitation leading to a quenching effect of LSPR was attributed to being responsible for the ultrafast optical switching (Figure 17a). Moreover, the plasmonic fiber tips with proper metallic nanostructures functionalized enable mode locking [93] and lasing [94]. Especially, Hua et al. proposed a plasmonic fiber tip for all-fiber Q-switched cylindrical vector lasers, where the fundamental mode was converted to the first-order mode in fiber (Figure 17b) [95]. The polarization-dependent plasmonic nanostructures were fabricated using FIB milling, and Q-switched azimuthally polarized beam and radially polarized beam were delivered at the wavelength of 1548.5 nm with pulse durations from ~7 to ~2 μs when pump power increases from 30 to 120 mW. The efficiency was up to 21%.

## 5. Conclusions and Outlook

The emergence of plasmonic fiber tips has been a milestone in the whole optical community, ranging from theoretical advancements and device constructions to application developments. The general advantages of plasmonic fiber tips originate from the combination of optical fiber tips with functional nanostructures. Firstly, optical fiber tips possess the characteristics of miniaturization, robustness, and “plug and play” operation. In addition, unlike other fiber components, fiber tips offer a unique planar platform for functional integrations. Secondly, growing nanofabrication accessibilities bring great opportunities to explore new functionalities and application scenarios of conventional optical fibers. Moreover, with the help of optical fibers, practical applications of functional nanostructures have witnessed critical progress so far.

At the same time as benefitting from the combined advantages of fiber tips and functional nanostructures, plasmonic fiber tips suffer from the initial drawbacks of fiber tips and metallic nanofabrication. For example, the main challenge is to develop a mature fabrication technique and system for the integration of plasmonic nanostructures on optical fiber end-facets, with improved versatilities, yields, reduced cost, technique complexity. According to the above review, transfer methods and beyond are the most promising approach. Moreover, due to the passive nature of typical optical fibers, the related plasmonic fiber tips are limited as isolated devices.

Considering the above conclusions, the following trends are worthy of attention in the future:

- New achievements of modified transfer methods are expected. In terms of classical nanofabrication tools, each technique presents some limitations, while transfer methods offer special opportunities to combine the advantages of separate methods. Collective modifications are able to provide flexibility and versatility aiming at specific applications.
- The majority of the plasmonic fiber tips suffer from the initial Ohmic loss of metals, which substantially weakens the signal intensity and broadens the resonance linewidth. Therefore, material adjustments or/and structure optimizations are essential to improve the performances of plasmonic fiber tips [10]. For instance, silver outperforms most metals in loss, while the oxidization issue of silver is stumbling. Most recently, we found that Tamm plasmon polaritons (cavity modes confined between a distributed Bragg reflector and a metallic layer) are promising to improve the quality factor of plasmonic fiber tip up to 100 [96].
- An as-yet-unrealized milestone for plasmonic fiber tips is to achieve active functionality. Current plasmonic fiber tips are static and suitable for one isolated application post-fabrication. Tunability would bring multi-functionality to the plasmonic fiber tips. Active materials could be chosen as the building blocks of plasmonic nanostructures [97] or modified onto plasmonic nanostructures to enable tunability for plasmonic fiber tips [98].
- Due to the complex nature of LSPR, wide ranges of material/fiber options, and diverse configurations of plasmonic nanostructures, the design of plasmonic fiber tips is always cumbersome. Machine learning has fundamentally changed many aspects of science and technology to date, facilitating the processing of large data that are difficult for manual modelling. Therefore, machine learning is beneficial to accurate and on-demand designs of plasmonic fiber tips for specific applications with unparalleled efficiencies.

## Data Availability

Not applicable.
